# Health Care Use and Costs for Participants in a Diabetes Disease Management Program, United States, 2007-2008

**Published:** 2011-04-15

**Authors:** Timothy M. Dall, Mary Roary, Wenya Yang, Shiping Zhang, Yiduo Zhang, David R. Arday, Cynthia J. Gantt, Yaozhu J. Chen

**Affiliations:** IHS Global Insight. At the time of the study, Mr Dall was affiliated with The Lewin Group, Falls Church, Virginia.; Department of Health and Human Services, Office of the Secretary, Washington, DC. At the time of the study, Dr Roary was affiliated with The Lewin Group, and Capt Gantt was affiliated with TRICARE Management Activity; The Lewin Group, Falls Church, Virginia; The Lewin Group, Falls Church, Virginia; The Lewin Group, Falls Church, Virginia; TRICARE Management Activity, Falls Church, Virginia; US Naval Hospital, Agana, Guam; Covance, Princeton, New Jersey. At the time of the study, Ms Chen was affiliated with The Lewin Group, and Capt Gantt was affiliated with TRICARE Management Activity.

## Abstract

**Introduction:**

The Disease Management Association of America identifies diabetes as one of the chronic conditions with the greatest potential for management. TRICARE Management Activity, which administers health care benefits for US military service personnel, retirees, and their dependents, created a disease management program for beneficiaries with diabetes. The objective of this study was to determine whether participation intensity and prior indication of uncontrolled diabetes were associated with health care use and costs for participants enrolled in TRICARE's diabetes management program.

**Methods:**

This ongoing, opt-out study used a quasi-experimental approach to assess program impact for beneficiaries (n = 37,370) aged 18 to 64 living in the United States. Inclusion criteria were any diabetes-related emergency department visits or hospitalizations, more than 10 diabetes-related ambulatory visits, or more than twenty 30-day prescriptions for diabetes drugs in the previous year. Beginning in June 2007, all participants received educational mailings. Participants who agreed to receive a baseline telephone assessment and telephone counseling once per month in addition to educational mailings were considered active, and those who did not complete at least the baseline telephone assessment were considered passive. We categorized the diabetes status of each participant as "uncontrolled" or "controlled" on the basis of medical claims containing diagnosis codes for uncontrolled diabetes in the year preceding program eligibility. We compared observed outcomes to outcomes predicted in the absence of diabetes management. Prediction equations were based on regression analysis of medical claims for a historical control group (n = 23,818) that in October 2004 met the eligibility criteria for TRICARE's program implemented June 2007. We conducted regression analysis comparing historical control group patient outcomes after October 2004 with these baseline characteristics.

**Results:**

Per-person total annual medical savings for program participants, calculated as the difference between observed and predicted outcomes, averaged $783. Active participants had larger reductions in inpatient days and emergency department visits, larger increases in ambulatory visits, and larger increases in receiving retinal examinations, hemoglobin A1c tests, and urine microalbumin tests compared with passive participants. Participants with prior indication of uncontrolled diabetes had higher per-person total annual medical savings, larger reduction in inpatient days, and larger increases in ambulatory visits than did participants with controlled diabetes.

**Conclusion:**

Greater intensity of participation in TRICARE's diabetes management program was associated with lower medical costs and improved receipt of recommended testing. That patients who were categorized as having uncontrolled diabetes realized greater program benefits suggests diabetes management programs should consider indication of uncontrolled diabetes in their program candidate identification criteria.

## Introduction

Disease management programs educate patients and teach them self-management skills that lead to more healthful lifestyle choices and appropriate use of health care services. These improvements can produce better health outcomes and reduced medical costs. The Disease Management Association of America describes type 2 diabetes as one of the chronic diseases best suited for disease management; most studies have found improved patient outcomes after participation in diabetes management programs (1-3).

TRICARE Management Activity, the health care benefits administrator for US military service personnel, covers 9.4 million beneficiaries (active-duty service members, family members, and retirees and their family members); approximately 225,000 beneficiaries who are aged 18 to 64 years and ineligible for Medicare have been diagnosed with diabetes (4). In June 2007, TRICARE Management Activity contracted with its 3 regional managed care support contractors to implement a diabetes management program for patients whose medical records indicated high use of health care services. To improve patient selection criteria for program eligibility, inform program structure and content, and evaluate diabetes management programs, we assessed TRICARE's disease management program to determine whether and how patient outcomes differed by need for diabetes management (defined by indication of uncontrolled diabetes preceding program participation) and by intensity of program participation (defined by active or passive participation).

## Methods

### Overview

Patients were eligible to participate in this voluntary, opt-out program if during the previous 12-month period they had any diabetes-related emergency department (ED) visits or hospitalizations, more than 10 diabetes-related ambulatory visits, or more than twenty 30-day prescriptions for diabetes drugs.

From June 2007 through September 2008, 37,370 patients were automatically selected for enrollment in this program; 11% opted out. Among program participants, approximately 25% chose to receive personalized telephone counseling. Services included a baseline assessment by telephone with a care manager (lasting 40-50 min); monthly telephone calls to educate patients and assess progress toward goals; educational materials (eg, pamphlets, videos, cookbooks); and newsletters and online materials. These patients were categorized as "active" participants. The remaining 75% of patients, categorized as "passive" participants, received newsletters but chose not to receive personalized counseling. All patients could contact care managers by telephone if they had questions about their diabetes, and they could access Internet-based educational resources. Additional information about TRICARE's disease management program is available elsewhere (5,6).

The voluntary, opt-out nature of this program, which based eligibility on high use of health care services, introduced 3 phenomena that we considered in the evaluation design. First, selection bias occurs when highly motivated patients are more likely to participate, participate at higher intensity, or more actively manage their disease. Second, regression to the mean occurs when program eligibility is determined by high use of health care services. That is, patient medical costs and health care use tend to decline in the period following program eligibility (even in the absence of a disease management program) when participants are identified on the basis of their high health care use. Third, the opt-out design does not allow for a concurrent natural comparison group to quantify program effect.

We identified a historical control group (HCG) and used this group's information to develop equations predicting outcomes for patients in the absence of a diabetes management program. To estimate program effect, we calculated the mean difference between patients' observed and predicted outcomes.

To create the analytical database, we used a unique patient identifier that linked medical and pharmacy claims from the Defense Health Information Management System Clinical Data Repository, patient characteristics from the Defense Enrollment Eligibility Reporting System, and disease management services received as indicated in the patient tracking information systems of the 3 disease management service providers. An institutional review board (Independent Review Consulting, Inc, Corte Madera, California) approved the study protocols.

### Outcome metrics

On the basis of a literature review and data availability, we chose indicators considered best practices for evaluating program performance (7-9). Indicators of health care use were number of ED visits, hospital inpatient days, ambulatory visits, and number of prescriptions. We used the medical component of the Consumer Price Index to calculate TRICARE’s medical costs in 2008 dollars (10). Because most diabetes-attributed costs stem from its complications and higher use of general medical services, we report both 1) diabetes-related costs and health care use and 2) total costs and health care use (excluding claims for injury, pregnancy, congenital abnormalities, and cancer) (11). Clinical indicators were the percentage of patients who annually received at least 1 hemoglobin A1c (HbA1c) test, dilated retinal examination, or urinary microalbumin test. Laboratory test results were unavailable.

### Evaluation sample

From the 37,370 patients who were eligible for the program, we excluded 1) 3,021 patients with fewer than 6 months’ program enrollment (for insufficient time to complete the program), 2) 449 patients not continuously eligible for TRICARE during the evaluation period, 3) 218 patients who became eligible for Medicare (for whom complete medical records were unavailable), 4) 154 patients who died or had HIV/AIDS or end-stage renal disease (ESRD), and 5) 3,924 opt-out patients. The final sample contained 29,604 patients; the median program tenure was 15 months (Figure).

**Figure:**
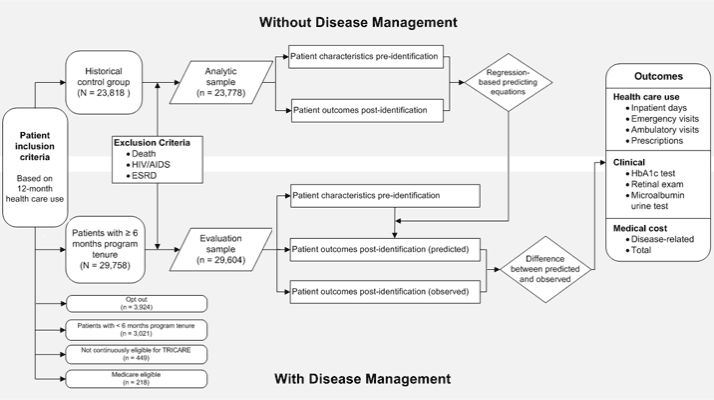
Evaluation model for the TRICARE diabetes disease management program, United States, 2007-2008. Abbreviations: ESRD, end-stage renal disease; HbA1c, hemoglobin A1c.

After TRICARE Management Activity identified candidates for the program, the disease management provider for each region contacted patients by letter and by telephone. Patients could choose to receive personalized counseling by telephone and mailing, to receive only the newsletters and other mailings, or to have no further contact with the program (ie, opt out). The disease management providers did not record patients' reasons for opting out. However, care managers said that opt-out patients' typical reasons for choosing not to participate is that they would be unable to complete the program because of impending military transfers and enlistment terminations, did not have diabetes, or previously participated in other diabetes management programs and did not believe they would benefit from participating in this program. Patients who opted out had similar demographic characteristics to those of participants but had slightly higher medical costs in the year preceding program eligibility.

The patient-level analytic file linked medical claims to program participation records. For each patient, we summarized health care use during 2 periods. The identification period was 1 year preceding the eligibility date; the postidentification period varied from 7 months to 15 months after the eligibility date. The outcome variables for each patient, standardized to a 12-month period, take into account a patient's program tenure. Although medical claims for participants were available through March 2009, to control for the time lag in claims processing and adjudication and to ensure we had complete claims data for participants, we allowed a minimum of 6 months from date of service to the final record extraction date and ended the observation period at September 30, 2008.

We categorized patients as having uncontrolled diabetes if they had at least 1 episode of care with the  diagnosis code for uncontrolled diabetes (*International Classification of Diseases, Ninth Revision*, 250.02, 250.03) during the year preceding program eligibility. Although lack of a medical claim with a diagnosis of uncontrolled diabetes does not necessarily mean a patient's glucose level was controlled, for discussion purposes we refer to these patients as having "controlled" diabetes.

### Historical control group

The HCG contained 23,818 patients who in October 2004 met the criteria used later to determine eligibility for the program established in June 2007 (Figure). After excluding patients who died or had HIV/AIDS or ESRD, the HCG analytic sample consisted of 23,778 patients. We used multivariable regression to assess the difference between patient health care use and costs, by patient characteristics, postidentification (November 2004 through October 2005) and from the eligibility determination period (November 2003 through October 2004).

We used Poisson regressions to analyze count variables such as inpatient days and ED visits, generalized linear models with gamma distribution to analyze health care costs, and logistic regressions to analyze dichotomous clinical variables such as having an HbA1c test during the year. Covariates included patient age group (18-34 y, 35-54 y, 55-64 y) and sex; TRICARE region; military service branch; number of ED visits, inpatient days, ambulatory visits, and 30-day prescriptions; indication of uncontrolled diabetes; Charlson comorbidity index score (12,13); and medical costs. Regressions to estimate diabetes-specific health care use and costs incorporate diabetes-specific health care use and cost covariates. We estimated separate regressions for 1) patients covered under the managed care plan and 2) patients in the preferred provider organization (PPO) plans.

Regression results, available from the authors on request, are consistent with expectations. For example, sicker patients (ie, those with a higher Charlson comorbidity score) in the November 2003 through October 2004 period had higher average health care use and costs in the November 2004 through October 2005 period. The goodness-of-fit measures for the regressions — based on root mean square error for continuous outcomes and receiver operating curve for dichotomous outcomes — suggest that the regressions are robust. We used the regression coefficients based on these HCG analyses to predict program participants' annual health care use and costs.

## Results

### Sample characteristics

Of the 29,604 participants in the analytic sample, 12,776 (43%) had indications of uncontrolled diabetes in the year preceding program start (Table 1). The rates of active participation were similar for patients with indications of uncontrolled (26%) and controlled (25%) diabetes. A higher proportion of active participants than passive participants were in the managed care plan and had higher preprogram rates of receiving an HbA1c test, retinal examination, and microalbumin urine test. They also had a higher Charlson comorbidity score and higher preprogram health care use and medical costs.

The HCG was similar to the program participant group in demographic characteristics, medical costs, and health care use. However, patients in the HCG had lower rates of receiving appropriate tests, particularly HbA1c and microalbumin urine tests.

### Health care use and costs

Because high health care use determined program eligibility, patient health care use and cost outcomes were higher in the year preceding program eligibility than in the year following eligibility (as extreme scores moved toward being less extreme). For the HCG, diabetes-related costs dropped by 28% and total costs dropped by 17% from the preidentification to postidentification period (Table 2). However, the decline was even larger for the program participants, who experienced 35% and 20% postprogram declines in annual diabetes-related and total costs, respectively, compared with the year preceding program eligibility. Before adjusting for case mix, these estimates suggest that program participants generated $353 per year less in diabetes-related costs and $408 per year less in total costs than did the HCG group. The decline in diabetes-related costs was largest for patients with prior indications of uncontrolled diabetes, and the decline in total costs was largest for patients with no prior indication of uncontrolled diabetes.

### Observed versus predicted outcomes

Program participants with prior indication of uncontrolled diabetes had higher observed diabetes-related costs per year (Table 3) and higher total costs per year (Table 4) than did patients with controlled diabetes. However, this outcome was expected, based on prediction equations generated from the HCG group, because patients with uncontrolled diabetes had higher Charlson comorbidity scores than did patients with no indication of uncontrolled diabetes in the year preceding eligibility.

The difference in observed and predicted outcomes (which controls for differences in case mix between subsets of program participants) suggests an average annual reduction in diabetes-related costs of $249 per person overall (Table 3). The reduction was largest among patients with a previous indication of uncontrolled diabetes, whether active ($388) or passive ($392), driven primarily by larger-than-average reductions in use of inpatient services; the smallest decline in average annual diabetes-related costs was for active participants with controlled diabetes ($67) (data not shown). Costs associated with the rise in diabetes-related ambulatory visits and receipt of tests partially offset medical savings associated with the modest decline in use of hospital services. Program participation was associated with receiving an annual HbA1c test, retinal examination, and microalbumin urine test compared with the year preceding enrollment; active participants experienced the greatest improvement.

The average per-person reduction in total costs was $783 per year (Table 4). The reduction was largest for active participants with prior indications of uncontrolled diabetes ($1,007) and smallest for passive participants with controlled diabetes ($577) (data not shown). Compared with passive participants, active participants experienced larger declines in inpatient days and ED visits and larger increases in ambulatory visits. For passive participants, medical savings appear to be primarily realized through reduced use of prescription drugs.

## Discussion

This study suggests that TRICARE's diabetes management program is associated with modest annual reductions in medical costs and improved receipt of recommended screening. Improvements were largest for active participants with prior indication of uncontrolled diabetes and smallest for patients with no prior indication of uncontrolled diabetes (whether active or passive participants). Active participants had greater improvements than passive participants in the proportion receiving appropriate screening. Increased costs for ambulatory care offset a portion of medical savings from reduced inpatient days and ED visits.

Our estimates of average annual medical savings among all program participants ($249 for diabetes-related costs and $783 for total costs) are within the range of estimates from other diabetes management programs. Published estimates for other programs, which may differ from this program in terms of population served and intensity of services, suggest an average annual per-patient savings of $626 in diabetes-related costs (with estimates ranging from a $2,787 loss to a $4,329 gain) (2,14-16). This average comes from randomized clinical trials, quasi-experimental, and pre-post studies (although the sample sizes in these studies are much smaller than for our study). Studies that used a quasi-experimental design similar to our use of the HCG estimated average per-patient savings to be approximately $1,292 per year (2).

A major contribution of our study is that patients with prior indications of uncontrolled diabetes appear to experience greater program benefits than do patients with no indication of uncontrolled diabetes in the 12 months preceding program eligibility. Patients with uncontrolled diabetes who want to improve their self-management skills (as indicated by active program participation) likely have the most to gain from an education-based diabetes management program.

Compared with passive program participation, active participation is associated with larger reduction in inpatient days and ED visits and higher rates of recommended screening. Active participants also experienced larger increases in ambulatory visits and smaller decreases in use of medications. These differences in outcomes by participation intensity were not as pronounced as expected. The voluntary nature of this program, in which patients could choose their participation intensity, warrants caution when making comparisons between active and passive participants. We controlled for differences between active and passive participants, such as active participants' slightly higher preprogram expenditures for diabetes-related health care services, by using prediction equations. In addition, we controlled for differences that likely correlated with patients' motivation to improve their health: active participants had higher rates of receiving at least 1 HbA1c test, retinal examination, and microalbumin urine test annually.

This study had several limitations. Patients could opt out of the disease management program, whereas the HCG had no opt-out component. Anecdotal evidence suggests that many who opted out would have been excluded from the HCG, for example, patients who would soon be leaving the Military Health System. Observed characteristics for the opt-out group were similar to those of program participants. The opt-out nature of this program also meant that there was no similar concurrent control group.

The HCG was part of our study design because comparing program outcomes for TRICARE's disease management program with outcomes from published literature would have posed multiple problems. These include the datedness of published findings and the differences in populations, diseases, program designs, and insurance benefits. Identifying an HCG that met the eligibility criteria for program participation within the TRICARE health plan limited these issues. By using prediction equations, we could control for differences between the HCG and program participants (17). A limitation of this approach is that secular trends continue to change health care delivery patterns. To mitigate the potential bias from secular trends we 1) chose an HCG that predated the disease manage program only slightly, 2) used patient preprogram characteristics to predict outcomes after program start, and 3) adjusted past health care costs to 2008 dollars.

Unobservable differences between active and passive participants may explain the smaller-than-expected differences in outcomes. Our study, for example, does not control for the possibility that patients received adequate diabetes counseling from other sources (eg, primary care provider or endocrinologist). Such information is unavailable for both the HCG and the program participants. If patients who already receive good diabetes counseling from other sources are more likely to be passive participants then our prediction equations could overestimate the program's effect on passive participants and underestimate the effect on active participants when comparing observed with predicted outcomes.

Another limitation is the inability to precisely identify patients whose diabetes is controlled. The unavailability of laboratory results (eg, HbA1c results) prohibited us from testing whether patients with extremely high glucose levels benefited from the program more than patients with moderately high glucose levels. Some patients with no diagnosis code for uncontrolled diabetes undoubtedly had uncontrolled diabetes, and some patients with a prior diagnosis of uncontrolled diabetes likely had their diabetes under control at the start of disease management. This inexactness in identifying patients with uncontrolled diabetes may reduce the estimates of program differences by uncontrolled status.

TRICARE's education-based disease management program was associated with modest reductions in annual medical costs and more appropriate use of health care services. Patients with prior indications of uncontrolled diabetes actively participated in the program at the same rate as patients with no indication of uncontrolled diabetes, and they appeared to benefit more from the program, leading to higher cost savings. This finding suggests that patients with indications of uncontrolled diabetes are strong candidates for participation in diabetes management programs.

## Figures and Tables

**Table 1:** Characteristics and Outcomes of Participants in the TRICARE Diabetes Disease Management Program, United States, 2007-2008

Characteristic/Outcome	Diabetes Status/Program Participation Status^a^				All, n = 29,604	Historical Control Group,^b^ n = 23,778

	Controlled		Uncontrolled			

	Active, n = 4,204	Passive, n = 12,624	Active, n = 3,332	Passive, n = 9,444		
**Age, mean (SD), y**	55.1 (8.0)^c^	53.0 (9.7)	53.9 (9.1)^d^	50.6 (11.5)	52.6 (10.1)	53.0 (9.9)
**Men, %**	40^c^	48	37^d^	44	44	44
**Region, %**						
North	37^c^	22	31^d^	20	24	31
South	47^c^	52	51	52	51	41
West	16^c^	26	18^d^	28	24	28
**Military branch, %**						
Army	41	39	38	39	39	39
Air Force	27	28	29	29	28	25
Navy	25	25	25	25	25	29
All other	7	7	8	7	7	7
**Managed care plan, %**	62^c^	57	72^d^	67	63	63
**Preventive care received in previous 12 months, %**						
HbA1c test	5^c^	43	71^d^	66	55	28
Retinal exam	23^c^	19	35^d^	30	25	23
Microalbumin urine test	32^c^	28	47	47	37	16
**Charlson comorbidity score,^e^ mean (SD)**	0.9 (1.2)^c^	0.8 (1.2)	1.3 (1.5)^d^	1.1 (1.4)	0.9 (1.3)	0.7 (1.2)
**Health care use, mean (SD)**						
No. of ED visits	1.6 (2.4)	1.6 (3.1)	2.1 (3.8)	2.1 (3.7)	1.8 (3.3)	1.8 (3.4)
No. of inpatient days	1.5 (5.0)	1.4 (5.8)	3.2 (9.2)	3.0 (8.9)	2.1 (7.3)	2.5 (8.1)
No. of ambulatory visits	22 (24)^c^	18 (22)	33 (29)^d^	27 (26)	23 (25)	22 (23)
No. of 30-day prescriptions	93 (57)^c^	77 (54)	104 (60)^d^	86 (56)	85 (56)	81 (55)
**Diabetes-related costs,^f^ mean (SD), $**	4,148 (5,042)^c^	3,607 (5,160)	7,398 (8,416)^d^	6,925 (8,482)	5,169 (6,972)	5,215 (7,738)
**Total costs,^f^ mean (SD), $ **	13,452 (19,663)^c^	11,104 (16,744)	20,387 (25,156)^d^	16,689 (21,770)	14,264 (20,181)	14,376 (21,907)

Abbreviations: SD, standard deviation; HbA1c, hemogloblin A1c; ED, emergency department.

a Controlled defined as not having an episode of care with the *International Classification of Diseases, Ninth Revision*, diagnosis code for uncontrolled diabetes (250.02, 250.03) during the year preceding program eligibility. Uncontrolled defined as having at least 1 such episode. Active defined as receiving personalized telephone counseling; passive defined as declining personalized counseling but receiving educational mailings.

b Refers to patients who in October 2004 met the criteria used later to determine eligibility for the diabetes disease management program established in June 2007.

c Significantly different from participants in the controlled, passive category at *P* < .05. Calculated by using a 2-tailed *t* test.

d Significantly different from participants in the uncontrolled, passive category at *P* < .05. Calculated by using a 2-tailed *t* test.

e An index of comorbid conditions; a higher score indicates a sicker patient (12).

f Adjusted to the medical component of the Consumer Price Index in 2008 (10).

**Table 2:** Health Care Costs, by Diabetes Status, Before and After Participation in the TRICARE Diabetes Disease Management Program, United States, 2007-2008^a^

Cost Category/ Diabetes Status^b^	Period	Historical Control Group (HCG),^c^ n = 23,778		Program Participants, n = 29,604		Difference Between HCG and Participants	

		Mean Per-Person Annual Cost,^d^ $	Pre-Post Change, %	Mean Per-Person Annual Cost,^d^ $	Pre-Post Change, %	$	%
**Diabetes costs**							
Controlled	Pre	3,720	−27.8	3,568	−34.6	−200	−6.8
	Post	2,687		2,335			
Uncontrolled	Pre	6,822	−27.4	6,627	−34.7	−427	−7.3
	Post	4,950		4,328			
All	Pre	5,215	−27.6	5,169	−34.6	−353	−7.1
	Post	3,777		3,378			
**Total costs**							
Controlled	Pre	11,239	−14.4	11,307	−19.3	−564	−4.9
	Post	9,620		9,124			
Uncontrolled	Pre	17,751	−19.4	16,957	−21.0	−120	−1.6
	Post	14,308		13,394			
All	Pre	14,376	−17.4	14,264	−20.4	−408	−3.0
	Post	11,879		11,359			

a Unadjusted for case mix.

b Controlled defined as not having an episode of care with the *International Classification of Diseases, Ninth Revision*, diagnosis code for uncontrolled diabetes (250.02, 250.03) during the year preceding program eligibility. Uncontrolled defined as having at least 1 such episode.

c Refers to patients who in October 2004 met the criteria used later to determine eligibility for the diabetes disease management program established in June 2007.

d Adjusted to the medical component of the Consumer Price Index in 2008 (10).

**Table 3:** Predicted and Observed Diabetes-Related Health Care Use and Costs for Participants in the TRICARE Diabetes Disease Management Program, United States, 2007-2008^a^

Outcome	Diabetes Status/Program Participation Status^b^				All, n = 29,604

	Controlled, n = 16,828	Uncontrolled, n = 12,776	Active, n = 7,536	Passive, n = 22,068	
**Observed outcome**					
Total costs,^c^ mean (SD), $	2,516 (3,633)	4,514 (6,685)	3,692 (4,626)	3,271 (5,467)	3,378 (5,269)
No. of inpatient days, mean (SD)	0.1 (1.2)	0.4 (2.8)	0.2 (1.4)	0.3 (2.2)	0.3 (2)
No. of ED visits, mean (SD)	0.2 (0.6)	0.3 (1.1)	0.2 (0.8)	0.2 (0.8)	0.2 (0.8)
No. of ambulatory visits, mean (SD)	3.8 (4.6)	6.6 (6.7)	5.7 (6.1)	4.7 (5.6)	5.0 (5.8)
No. of 30-day prescriptions, mean (SD)	19 (15)	23 (15)	23 (15)	20 (15)	21 (15)
Received HbA1c test, %	53	71	69	58	61
Received retinal exam, %	22	31	32	24	26
Received microalbumin urine test, %	33	47	44	37	39
**Predicted outcome**					
Total costs,^c^ mean (SD), $	2,657 (1,676)	4,905 (3,032)	3,900 (2,548)	3,534 (2,622)	3,627 (2,608)
No. of inpatient days, mean (SD)	0.2 (0.2)	0.5 (1.2)	0.3 (0.7)	0.3 (0.9)	0.3 (0.8)
No. of ED visits, mean (SD)	0.1 (0.1)	0.3 (0.5)	0.2 (0.3)	0.2 (0.3)	0.2 (0.3)
No. of ambulatory visits, mean (SD)	3.6 (2.1)	6.5 (2.8)	5.3 (2.9)	4.7 (2.7)	4.9 (2.8)
No. of 30-day prescriptions, mean (SD)	21 (12)	24 (11)	24 (12)	21 (12)	22 (12)
Received HbA1c test, %	50	69	63	57	58
Received retinal exam, %	20	27	26	23	23
Received microalbumin urine test, %	28	41	35	33	33
**Difference in observed and predicted outcomes^d^**					
Total costs,^c^ $	−141	−391	−208	−263	−249
No. of inpatient days	−0.03	−0.07	−0.09	−0.03	−0.05
No. of ED visits	0.01	0.03	0.002	0.03	0.02
No. of ambulatory visits	0.14	0.11	0.42	0.02	0.13
No. of 30-day prescriptions	−1.3	−0.9	−0.4	−1.3	−1.1
Received HbA1c test, %	3	2	6	1	2
Received retinal exam, %	2	3	7	1	2
Received microalbumin urine test, %	6	7	9	5	6

Abbreviations: SD, standard deviation; ED, emergency department; HbA1c, hemoglobin A1c.

a Adjusted for case mix.

b Controlled defined as not having an episode of care with the *International Classification of Diseases, Ninth Revision*, diagnosis code for uncontrolled diabetes (250.02, 250.03) during the year preceding program eligibility. Uncontrolled defined as having at least 1 such episode. Active defined as receiving personalized telephone counseling; passive defined as declining personalized counseling but receiving educational mailings.

c Adjusted to the medical component of the Consumer Price Index in 2008 (10).

d All differences between observed and predicted outcomes were significant at *P* < .05, calculated by using a paired *t* test. Differences may not be exact because of rounding.

**Table 4:** Predicted and Observed Total Health Care Use and Costs for Participants in the TRICARE Diabetes Disease Management Program, United States, 2007-2008^a^

Outcome	Diabetes Status/Program Participation Status^b^				All, n = 29,604

	Controlled, n = 16,828	Uncontrolled, n = 12,776	Active, n = 7,536	Passive, n = 22,068	
**Observed outcome**					
Total costs,^c^ mean (SD), $	9,619 (17,322)	13,650 (22,073)	12,922 (19,115)	10,825 (19,757)	11,359 (19,616)
No. of inpatient days, mean (SD)	1.1 (6.3)	1.9 (8.6)	1.4 (7.1)	1.4 (7.5)	1.4 (7.4)
No. of ED visits, mean (SD)	1.0 (2.8)	1.3 (3.9)	1.1 (3.4)	1.1 (3.3)	1.1 (3.3)
No. of ambulatory visits, mean (SD)	17 (21)	24 (26)	23 (25)	19 (23)	20 (23)
No. of 30-day prescriptions, mean (SD)	78 (53)	87 (55)	94 (56)	77 (53)	82 (54)
**Predicted outcome**					
Total costs,^c^ mean (SD), $	10,255 (7,206)	14,627 (9,776)	13,820 (9,152)	11,568 (8,445)	12,142 (8,686)
No. of inpatient days, mean (SD)	1.1 (1.5)	2.0 (2.8)	1.6 (2.2)	1.5 (2.1)	1.5 (2.2)
No. of ED visits, mean (SD)	0.9 (1)	1.3 (1.4)	1.1 (1.2)	1.0 (1.2)	1.1 (1.2)
No. of ambulatory visits, mean (SD)	16 (11)	22 (12)	21 (13)	18 (11)	19 (12)
No. of 30-day prescriptions, mean (SD)	84 (44)	92 (45)	99 (44)	84 (44)	87 (45)
**Difference in observed and predicted outcomes^d^**					
Total costs,^c^ $	−636^e^	−977^e^	−898^e^	−743^e^	−783^e^
No. of inpatient days	−0.03	−0.15^e^	−0.2^e^	−0.03	−0.1
No. of ED visits	0.1^e^	0.1	−0.04	0.1^e^	0.1^e^
No. of ambulatory visits	0.8^e^	1.2^e^	1.8^e^	0.7^e^	1^e^
No. of 30-day prescriptions	−6^e^	−5^e^	−4^e^	−6^e^	−6^e^

Abbreviations: SD, standard deviation; ED, emergency department; HbA1c, hemoglobin A1c.

a Adjusted for case mix.

b Controlled defined as not having an episode of care with the *International Classification of Diseases, Ninth Revision*, diagnosis code for uncontrolled diabetes (250.02, 250.03) during the year preceding program eligibility. Uncontrolled defined as having at least 1 such episode. Active defined as receiving personalized telephone counseling; passive defined as declining personalized counseling but receiving educational mailings.

c Adjusted to the medical component of the Consumer Price Index in 2008 (10).

d Differences between observed and predicted outcomes may not be exact because of rounding.

e Difference between observed and predicted outcomes significant at *P* < .05, calculated by using a paired *t* test.

## References

[1] Knight K, Badamgarav E, Henning JM, Hasselblad V, Gano AD, Ofman JJ (2005). A systematic review of diabetes disease management programs. Am J Manag Care.

[2] Goetzel RZ, Ozminkowski RJ, Villagra VG, Duffy J (2005). Return on investment in disease management: a review. Health Care Financ Rev.

[3] Sidorov J, Shull R, Tomcavage J, Girolami S, Lawton N, Harris R (2002). Does diabetes disease management save money and improve outcomes? A report of simultaneous short-term savings and quality improvement associated with a health maintenance organization-sponsored disease management program among patients fulfilling health employer data and information set criteria. Diabetes Care.

[4] (2009). Basic facts of the military health system/2008 MHS stakeholders report.

[5] Dall TM, Askarinam-Wagner R, Zhang Y, Yang W, Arday DR, Gantt CJ (2010). Outcomes and lessons learned from evaluation of TRICARE's disease management programs. Am J Manag Care.

[6] Yang W, Dall TM, Zhang Y, Hogan PF, Arday DR, Gantt CJ (2010). Disease management 360°: a scorecard approach to evaluating TRICARE's programs for asthma, congestive heart failure, and diabetes. Med Care.

[7] American Diabetes Association (2009). Standards of medical care in diabetes — 2009. Diabetes Care.

[8] American Healthways, Johns Hopkins (2003). Standard outcome metrics and evaluation methodology for disease management programs. American Healthways and Johns Hopkins Consensus Conference. Dis Manag.

[9] (2004). Disease management program evaluation guide.

[10] Databases, tables, and calculators, by subject. All urban consumers.

[11] American Diabetes Association (2008). Economic costs of diabetes in the U.S. in 2007. Diabetes Care.

[12] Charlson ME, Pompei P, Ales KL, Mackenzie CR (1987). A new method of classifying prognostic comorbidity in longitudinal studies: development and validation. J Chronic Dis.

[13] Deyo RA, Cherkin DC, Ciol MA (1992). Adapting a clinical comorbidity index for use with ICD-9-CM administrative databases. J Clin Epidemiol.

[14] Beaulieu ND, Fund C (2003). The business case for diabetes disease management at two managed care organizations: a case study of HealthPartners and Independent Health Association.

[15] Snyder JW, Malaskovitz J, Griego J, Persson J, Flatt K (2003). Quality improvement and cost reduction realized by a purchaser through diabetes disease management. Dis Manag.

[16] Berg GD, Wadhwa S (2002). Diabetes disease management in a community-based setting. Manag Care.

[17] Cousins MS, Liu Y (2003). Cost savings for a preferred provider organization population with multi-condition disease management: evaluating program impact using predictive modeling with a control group. Dis Manag.

